# Book Reviews

**Published:** 1830-03

**Authors:** 


### CRITICAL ANALYSES.

Quæ laudanda forent, et quæ culpanda, vicissim

Illa, prius, cretâ; mox hæc, carbone, *notamus*.—PERSIUS.

*Researches principally relative to the Morbid and Curative Effects of Loss of Blood.* By MARSHALL HALL, M.D. F.R.S.E. &c. &c.—8vo. pp. 303. Seeley and Burnside, London, 1830.

IN our last Number we gave an analysis of the first part of this ingenious and interesting work. We shall now proceed to submit to our readers a condensed account of the second part, the object of which is to treat of bloodletting as a remedy; to prescribe and to limit its use, and to guard against its undue employment. It is impossible that more important points for consideration can occupy the time of an author. Dr. HALL's attempt is at least creditable, and, if it be attended with success, even partial success, it must prove of the utmost benefit in our management of disease. A quarter of a century ago, debility occupied every man's mind, and the profession were deterred from the use of the lancet in cases in which we now know it was imperatively required. A reaction of opinion took place, and became excessive, and led to an immoderate employment of bloodletting. Both these extremes of opinion were fatal. From the influence of the former doctrine, inflammation was frequently allowed to run on, and to destroy its victim. The second error is more often fatal, by inducing irremediable exhaustion. How then shall we avoid extremes, alike so replete with evil? This important question Dr. Hall endeavours to answer in the second part of his work.

"It is one of the most remarkable facts in physic, that if several patients of similar strength and constitution, but affected by dissimilar diseases, be respectively placed in the erect position and bled to deliquium, they will be found to have lost very various quantities of blood. I have known a patient, not apparently very feeble, faint on losing four ounces of blood; and I have known patients bear to lose fifty, sixty, and even seventy ounces of blood without syncope.

"This fact, plain and simple as it is, with its rationale and practical applications, has, I think, been greatly overlooked.

"Its rationale is to be found, I believe, in connexion with an equally interesting fact, that different diseases induce in the constitution different powers or susceptibilities in regard to the effects of loss of blood. Each disease appears, indeed, to possess its own peculiar and intrinsic virtue in this respect. This is determined by placing the patient perfectly erect, and bleeding to incipient syncope: the quantity of blood which flows is the measure of the protective influence of the disease in one class of cases, and of its influence in superinducing a susceptibility to the effects of loss of blood in the other.

"An interesting scale of diseases may be formed representing these properties. It would begin with congestion of the head, or tendency to apoplexy; inflammation of the serous membranes, and of the parenchymatous substance of various organs, would follow; then acute anasarca; and, lastly, inflammation of the mucous membranes. This part of the scale would be divided from the next by the condition of the system in health. Below this would be arranged fever, the effects of intestinal irritation, some cases of delirium, reaction from loss of blood, and disorders of the same class with hysteria, dyspepsia, chlorosis, and cholera morbus.

"Persons in health and of moderate strength will generally faint, if bled in the erect posture, on taking fifteen ounces of blood. I have known seventy ounces to be taken in the sitting posture, in the tendency to apoplexy, without syncope; but the case is an extreme one. Patients with pleuritis or pneumonia frequently lose thirty-five ounces of blood without fainting. In bronchitis, little more is borne to be lost than in health. A stout person in fever will frequently faint on losing ten, twelve, or fourteen ounces of blood. In intestinal irritation, with urgent symptoms even, the abstraction of nine or ten ounces of blood will generally induce deliquium. In delirium tremens, or puerperal delirium, the patient soon faints from loss of blood. The same thing is still more observed in those cases of violent reaction which arise from loss of blood itself. In dyspepsia, hysteria, and chlorosis, the susceptibility to syncope from loss of blood is very great; and I have known a patient, of good strength, affected with cholera, faint on taking four ounces of blood, although she had shortly before borne to lose nearly twenty ounces without faintishness, under the influence of inflamed mamma.

"I imagine that the rationale of this fact will be found in the obvious difference in the nature of these diseases. In all those cases in which the circulation of the heart and larger arteries alone is affected, and especially in such as involve irritation or exhaustion, there is early syncope on taking blood. But in such cases as consist in an affection of the capillary circulation, and especially such of these as affect the head, it requires the abstraction of much blood to induce deliquium. Syncope is prevented by the influence exerted by this state of the capillary circulation over that of the heart and larger arteries, and over the whole system, and especially over the circulation within the brain; and it does not entirely subdue the morbid action of the capillary vessels even when induced. To induce syncope in pure fever, we have then but to subdue the state of reaction in the heart and larger arteries. In inflammation, we have not only to do this, but to overcome the influence of a permanent morbid action of the capillaries: this is especially observed in inflammation of the serous membranes and within the head.

"The practical application of this fact consists chiefly in its affording a rule for bloodletting in all cases in which this measure is required to be fully instituted; a guard against undue bloodletting, both in this and some other cases; and a source of diagnosis.

"The quantity of blood which flows when a patient requiring full bloodletting is placed upright and bled to deliquium, seems accurately proportionate to the exigencies of the case. In inflammation much blood should be taken, and much blood will flow before deliquium is induced: in irritation little blood should be drawn, and there is early syncope from bloodletting. The quantities are even accurately suited, not only to the exigencies of the disease, but to the powers of the system; at least, so it appears to me from considerable experience.

"The rule is suited also to the degree and the duration of the disease; for, with each of these, its influence in inducing tolerance or intolerance of loss of blood is respectively augmented.

"It is not less adapted to those most frequent of all events, mixed cases. Inflammation and irritation may be conjoined: for example, there may be mere nephralgia, or absolute nephritis, from calculus, or a mixed case involving both. There may be mingled intestinal irritation and inflammation. In each of these circumstances, the rule for bloodletting which I have proposed adapts itself accurately to the demands of these various morbid affections, and to the actual strength and condition of the general system." (P. 175.)

Dr. Hall proceeds to state that, as in inflammation we *must*, so we *may* bleed freely; whilst in other cases bloodletting, as it is not so required, so it is not so *borne*. These points are determined first by the diagnosis, but secondly by the ready or tardy induction of syncope on taking blood in the perfectly erect position. Dr. Hall observes further, that in all cases in which there is early syncope from blood-letting, the secondary or more remote effects of loss of blood are most apt to supervene: in such cases, too, there is the greatest danger of the sinking or more sudden failure of the vital powers. The rule proposed becomes in its turn, too, diagnostic; great tolerance of loss of blood denotes inflammation; great susceptibility to its effects, diseases of another and very opposite character.

These observations are followed by a valuable contribution from the pen of our talented correspondent, Mr. Heming, copied from the Medical Gazette. Mr. Heming's experience confirms the statements of Dr. Hall in every respect. This gentleman has also brought together several cases from various authors illustrative of the same views.

We are next led to the consideration of "some diseases in their relation to loss of blood." Under this head the author passes in review fever, inflammation, irritation, accidents, and operations.

"Fever seems to differ from inflammation in being an affection of the whole nervous and vascular systems; in inflammation there is an affection of these systems in one part or organ.

"There is another difference between these two diseases: fever appears to consist in an affection of the nervous system and of the heart and larger arteries, the capillary vessels being only affected as an extension of this morbid state. In inflammation there is, according to the experiments of Dr. Wilson Philip and Dr. Hastings, a primary affection of the capillary vessels, consisting in enlargement of their diameter, and a slower movement of more numerous globules of the blood. A consequence which flows from this view of the subject is, that to subdue momentarily the state of fever, we have only to subdue the augmented action of the heart and larger arteries; but as the capillary circulation is less immediately under the influence of the heart, the action of the former may be subdued, whilst a morbid state of the latter may be continued with comparatively little change.

"It is upon this principle, I believe, that a fact is to be explained, which will be frequently adverted to in this work, that syncope is more readily produced by the abstraction of blood in pure fever, and in other diseases consisting alike in the state of the heart and larger arteries, than in pure inflammation, consisting in a peculiar condition of the capillary vessels, more permanent and less under the influence of the general circulation.

"In the former case, syncope is the simple effect of depriving the heart and arteries of their accustomed stimulus, and this probably under circumstances of augmented susceptibility of the nervous system to impressions of this kind; in the latter, although blood may be taken, and the action of the heart and arteries be thus subdued, yet, from a less degree of susceptibility of the nervous system, and from the unsubdued morbid action of the capillaries, acting as it were as a permanent stimulus to the general system, syncope is not so soon induced by the abstraction of blood. But whatever the explanation of this fact may be, the fact itself is, I think, established upon the sure ground of multiplied experiment.

"There are three circumstances in fever which should lead to the use of the lancet. The first is excessive reaction of the vascular system; the second, much excitement of the nervous system, especially violent delirium; and the third, and the most imperative, the existence of local inflammation. Each of these cases will require a few observations.

"In excessive vascular reaction, bloodletting is of the most essential service, especially early in the disease. The quantity of blood which should be taken must depend upon many circumstances, as the strength of the patient, the stage of the disease, the character of the epidemic. But the limit beyond which it would be dangerous to go is, I think, clearly marked out by the degree of susceptibility to the effects of loss of blood, denoted by the tendency to syncope on abstracting blood pretty freely in the erect posture. But, as I shall recur to this question, I would only repeat in the present place, that the susceptibility to the effects of loss of blood is far greater than in inflammation. I have known very stout persons, in the strong reaction of fever, faint on withdrawing four, six, eight, ten, and twelve ounces of blood in the erect posture.

"The same observation may be made in regard to great nervous excitement denoted by delirium. To abstract a moderate quantity of blood, does great good; but to bleed too freely, is dangerously to depress the powers of life. In this case, as in the last, the patient may safely be placed in the erect posture, and bled to incipient syncope, if it be a first bloodletting and early in the disease.

"But the most marked difference in regard to the powers of supporting the loss of blood, is superinduced by the addition of a local inflammatory affection to the original disease. The patient immediately becomes less prone to faint on being bled. It will be obvious how important it would be to establish this point accurately by an ample collection of facts, and thus to trace it in its reference to practice. It appears to me, from what I have hitherto ascertained, that there is, in every instance, a strict alliance between the degree of tolerance of loss of blood and the exigencies of the cure." (P. 197.)

Upon the important subject of "irritation," Dr. Hall offers many original and very ingenious remarks.

"I proceed to notice a morbid affection of very frequent occurrence, and with which the profession generally appear to me still to be totally unacquainted. This statement will not be deemed too strong, if I am enabled to show that there is a series of cases, not generally distinguished from certain inflammations, and yet very different in their nature, and especially in their reference to the effects of loss of blood.

"The cases to which I allude resemble, in their symptoms, the most acute forms of arachnitis, pleuritis, and peritonitis, but especially arachnitis. Yet, instead of possessing the power of resisting the effects of loss of blood belonging to inflammation, there is the utmost degree of susceptibility to those effects. In the former cases, thirty, forty, and even fifty ounces of blood may flow before the slightest deliquium is observed: in the latter there is frequently the most perfect syncope on abstracting nine or ten ounces of blood.

"The irritation of a calculus in the ureter, or in the hepatic duct, is well known to occasion a remarkable sympathetic affection of the stomach. The introduction of a bougie into the urethra sometimes induces rigor and a complete paroxysm of fever. Uterine irritation is not less frequently the cause of extraordinary effects upon the system generally, and upon various organs.

"But, of all the sources of sympathetic morbid affections, irritation in the stomach and bowels appears to be the most common, and certainly not the least important. Indigestible substances taken, and disordered feculent matter retained, are the frequent sources of that combined affection of the head and the stomach, termed sick headach.

"If such effects of local irritation upon distant organs, then, be universally known and admitted, it cannot be considered as extraordinary that others less recognised should exist. But such a point is to be established by facts, not by argument. I proceed, therefore, to detail some cases, which will, I doubt not, if perused with attention and without prejudice, fully convince the reader of the occurrence of a form of disorder hitherto overlooked, or mistaken for other diseases, but very distinct, and very important to be distinguished.

"The most frequent cause of this affection is a disordered state of the contents of the colon; the next is, some indigestible substance taken into the stomach. But as the mere presence of a calculus in the ureter is not always sufficient alone to induce an attack of pain and vomiting, so a deranged condition of the intestinal contents will not, alone, induce an attack of the morbid affection which I am about to describe: in general, some superadded cause, some shock sustained, or some effort made by the system, is necessary to rouse into activity the cause of irritation, otherwise dormant. In the same manner, indigestible substances may frequently be taken, when the health is unimpaired, with impunity; but, if the system be under the influence of shock, or effort, or of nervous or vascular excitement or exhaustion, a cause of disorder which might have been inert in other circumstances, proves of frightful activity.

"The effects of intestinal or nervous irritation, are chilliness, varying from coldness of the extremities to extreme rigor, followed by great heat of the surface, and symptoms resembling those of arachnitis or peritonitis, singly or successively, in their most acute forms, but especially arachnitis; more rarely there is pain resembling that of pleuritis; more rarely still, a peculiar pain passing along one side of the neck to the shoulders; and occasionally, generally after bloodletting, there is palpitation of the heart.

"It must be regarded as extraordinary that such marked affections have not been discriminated, and traced to their proper source. But I am persuaded that they are, to this day, confounded with inflammation of the organs chiefly affected, to the great injury, and even danger, of the patient. It is, indeed, extraordinary how slow the human mind is to receive new impressions, even of the truth, wedded as it usually is to first opinions.

"These observations apply particularly to that form of this affection which resembles arachnitis. There are few who distinguish it from arachnitis itself. I have, however, witnessed some very interesting scenes, and not less interesting convictions of the truth of the views which I have taken of this subject, in cases which have occurred in the persons or in the families of medical gentlemen themselves. Two of these cases I propose to detail briefly; premising, that the mere fact of the most acute symptoms, resembling those of arachnitis or peritonitis, having yielded without the abstraction of blood altogether, or without a shadow of that degree of bloodletting which is absolutely necessary for the cure of these inflammatory diseases, is alone sufficient to convince us that there is a case of morbid affection, resembling in its symptoms, but differing in its nature and treatment, from those diseases." (P. 210.)

We pass on to the discussion of the "due institution of bloodletting," the aim, and end, of our author's labours. Under this head Dr. Hall treats of *early* bloodletting; of a *first* bloodletting; of the *repetition* of the bloodletting; of bloodletting as a preventive of inflammation; of *late*, and of local bloodletting.

"Most diseases may, indeed, be divided into the stages, 1, of accession; 2, of full developement; 3, of disorganization of the part or parts affected; and 4, of deterioration or failure of the powers of the general system. It is very essential to bear this view in mind, whenever we may be required to determine the question of bloodletting. It will guide us far better than days or dates. If the disease be formed, and not merely expected, the earlier the lancet is used the better. If it be fully developed, bloodletting is still more required, and even better borne. It is when disorganization is great, and the powers of the system are shaken, that it requires the utmost caution and skill to conduct the treatment of the case.

"Early in the disease, a single bloodletting to syncope will often prove sufficient for the cure. If this remedy be employed later, it will usually be necessary and safe to repeat it." (P. 269.)

Upon the question of a first bloodletting, Dr. H. observes,

"The necessity and propriety of a first bloodletting must be determined by the diagnosis of the disease, and by a due estimation of the powers of the patient.

"In the case of inflammation, no one would think of trusting the safety of the patient to any other remedy than bloodletting; and in the case of irritation, bloodletting, although a subsidiary, may still be a useful remedy.

"The propriety of the measure having been thus determined, the next question is that of the due and proper mode of its administration. What quantity of blood should be taken? This question must involve the consideration of the nature, stage, and degree of severity of the disease, and of the strength, and the greater or less power or susceptibility in regard to the effects of loss of blood, of the patient. How difficult must it frequently be accurately to determine these points! This can only be done in many cases, indeed, by watching the effects of the loss of blood as it flows: and yet the usual mode of proceeding, is to prescribe the quantity of blood to be drawn, and forthwith to leave the patient in the hands of one from whom, however competent, the right, or at least the freedom, of judgment is thus preposterously taken." (P. 272.)

We have known many cases in which, if the following very important practical remark had been known or attended to, patients would probably have escaped from lingering and painful diseases.

"In determining the question of the propriety of a repetition of bloodletting, many circumstances require to be considered. But I would remark, in the first place, that if much blood have flowed at the first bloodletting, it must be taken as indicating the necessity for an early repetition of our visit at least; for it will also, unless the symptoms have been greatly subdued by it, indicate the necessity for a prompt repetition of the remedy." (P. 279.)

The author's remarks on bloodletting as a preventive of inflammation merit the attention of every practitioner.

The chapter "on bloodletting in infancy and childhood" is also replete with valuable practical comments.

"Not the least interesting application of the rule which I have proposed to guide and govern us in the use of bloodletting, is its use in the treatment of the diseases of infancy and childhood.

This tender age is far more liable than later years, both to the insidious and the sudden fatal effects of loss of blood; it therefore requires to be viewed with still greater care and watchfulness.

"The modes of general bloodletting employed in infancy and childhood are, leeching, cupping, and venesection.

"I must first, once for all, protest against the usual plan of applying leeches in infancy, and allowing the bites to continue to bleed. Nothing can be more indefinite; nothing more replete with danger. Most of all, it is dangerous to apply leeches late at night: the bleeding may go on unobserved and unsuspected, and precipitate the little patient into a state of irremediable sinking.

"The proper mode of abstracting blood in infants or children, whether by leeches, cupping, or venesection, is to place the little patient upright, and watch the countenance. On the very first indication of pallor or faintishness, the flow of blood must be stopped.* For this purpose the leeches, or the cupping glasses, are to be removed, or the vein secured." (P. 291.)

The vast importance of the subjects to which Dr. Hall directs the attention of the profession, is too obvious to be formally insisted upon; and we are confident that they who will attentively consider the doctrines inculcated in the volume we have just noticed, will be inclined with us to give it a high rank in the medical literature of the present day. We are gratified to find that a second edition of Dr. Hall's Treatise on Diagnosis is preparing for publication.

---

*A Treatise on Fever.* By Southwood Smith, M.D. Physician to the London Fever Hospital.—8vo. pp. 436. London: Longman, 1830.

*Clinical Illustrations of Fever; comprising a Report of the Cases treated at the London Fever Hospital,* 1828-1829. By Alexander Tweedie, M.D. Member of the Royal College of Physicians of London, Physician to the Fever Hospital, &c.—8vo. pp. 204. London: Whittaker, 1830.

A retrospective view of the subject of fever might easily have been compiled, and would have afforded us an opportunity of displaying our research; but we believe such a labour would impart neither amusement nor instruction to our readers. The most superficial medical student cannot be ignorant of the chaos of conflicting opinions that have divided the most eminent authorities upon this

* This may be done, in the case of a leech-bite or of venesection, if necessary, by taking up a small portion of the integuments by a mere stitch with a needle and silk.

important subject. Fever, indeed, has been to physicians a Gordian knot, which has frequently been rudely and hastily divided, but which has never been satisfactorily disentangled. We shall not even endeavour—*tantas componere lites,* but proceed at once to an analysis of the works before us. If we indulge in a longer article than usual, we are certain that our readers will freely admit our excuse, on account of the great interest which appertains to the subject discussed, and the strong claims which the authors have to our very deliberate attention. There is but one point respecting fever concerning which there is no disagreement. By all a knowledge of its phenomena, and of the influence which certain modes of treatment exert in modifying or subduing them, is deemed absolutely necessary for every practitioner who would perform his duties with credit to himself or advantage to his patients. In our notice of the volumes before us, we shall place in contact the opinions of the authors when they support each other, as well as when any difference of opinion exists between them. By this plan our readers will be put in possession of the pathological or practical points which are confirmed by both, and they will at the same time perceive any discrepancies which may obtain between two able and careful observers, who write from the same field of experience. It may be proper to observe, that we have placed the works at the head of this article in the order in which we received them, and that the one does not take precedence of the other from any acknowledged and evident superiority.

Dr. Smith informs us in his preface, that his work is wholly of a practical nature; its object is to ascertain the real phenomena of fever, and the most safe and effectual treatment of the disease. On looking over the account which he has given of the phenomena, he finds that he has omitted to mention the peculiar odour which belongs to a fever patient. "It is so characteristic, that a person familiar with the disease might in many cases be able to pronounce, merely from the odour of the effluvia that arises from the body, whether the disease were fever."

Dr. Tweedie also introduces us to his work with the assurance that he has confined himself as much as possible to facts, and if at any time he has been led into digression, it has been solely with the object of elucidating some point of practical importance. The practical inquirer need not apprehend, then, that he will be involved in the mazes of speculative fancies by either of our authors. He will sit down to the study of their volumes with the certainty of deriving from them useful instruction.

On the appointment of Dr. Smith to the office of physician to the London Fever Hospital, it was stated to him that, among the objects contemplated by the establishment of this institution, two things were conceived to be of paramount importance: first, the accumulation of facts by which the true nature of fever might be more certainly ascertained; and secondly, the cautious trial of remedies by which a more sure and successful mode of treating this fatal disease might be discovered. During his connexion with the hospital, he has faithfully endeavoured to keep these laudable and important objects in view.

The London Fever Hospital is capable of receiving sixty-two patients. In most seasons of the year its wards are full, and often, observes Dr. Smith, there are numerous applications for admission, which cannot be received for want of room. From six to seven hundred patients annually pass through the wards. Dr. Tweedie states that the total number of cases rejected in 1826 for want of accommodation in the hospital, was upwards of seven hundred. In a country where the hand of charity is so liberally opened, it is astonishing that a more capacious institution for fever patients has not been established.

After some preliminary observations in proof of the necessity of still further investigating the subject of fever, on account of the perplexing diversity of opinion respecting both its pathology and treatment, Dr. Smith gives a rapid sketch of the ancient and modern doctrines relative to the nature and seat of the disease. The slightest glance, he says, at the history of these doctrines from remote antiquity, and more especially a consideration of the variety, and even the contrariety, of the opinions respecting both the nature and seat of fever which are received at the present day, but too clearly show that, if the ancients were in error, there cannot be many points with regard to which the moderns are right, since there is scarcely one in which they are agreed. We must presume our readers to be acquainted with the doctrines of Cullen, Brown, Stoker,* Burne, Clanny, Clutterbuck, and Broussais, and shall,

* It was originally our intention to have given a sketch of Dr. Stoker's last work together with the analysis of Dr. Smith's and Dr. Tweedie's, but as we found, upon perusing it, that neither the pathological doctrines it contains, nor the plan of treatment described in it, could be applied to the fevers of London, we have postponed our notice of it for a future Number.—Editor.

therefore, pass over the brief notice bestowed upon them by Dr. Smith. The comments he makes upon the errors which are common to all these theorists, are eloquently and acutely penned.

"The believers in debility derive their notion of the whole disease from the phenomena which occur in the first and last stages only: in these, it is true, they may find abundant evidence of debility; but then they overlook the intermediate stage, in which there are generally the most unequivocal indications of increased sensibility in the nervous and increased action in the vascular systems: in this manner they characterize the disease by what appertains only to certain stages of it. Again, when they contend that debility is not only the essence of fever in general, but is really characteristic of every type of it, they affirm what is indisputable of fevers in particular seasons, in particular climates, or in particular constitutions; but beyond this their generalization cannot be extended: in this manner they assign to the genus what belongs only to the species. And when Cullen goes on to affirm that the proximate cause of all the morbid phenomena is a 'spasm of all the extreme vessels,' he commits the additional and more palpable, but not less common error, of assigning as an undoubted fact, as a real and ascertained occurrence, what is only a conjecture, and for which there is not, and for which he does not even attempt to adduce, the shadow of evidence.

"Precisely similar to this is the error of those who for the most part belong to the same school, and who attribute the essence of fever to a morbid condition of the blood. The blood may be diseased in fever; but if it be so, these writers do not *know* it, or at least they do not adduce any evidence that they are in possession of such knowledge; they do not appear so much as to have questioned chemistry; at all events, it is certain that they have hitherto received no satisfactory answer. There is no evidence on record that the alleged determination of the blood takes place in every type and every degree of fever; and if there were, it would still be but one event among many, and one that occurs late in the series, and therefore could possibly be nothing more than an effect.

"In like manner, those who maintain that inflammation of the brain is the sole cause of fever, assume as an established and admitted fact, the universal and invariable existence of inflammation of the brain in this disease. Inflammation of the brain, without doubt, is demonstrable of many individual cases, and of some whole types; but beyond this there is no proof that the generalization can be carried. The evidence, indeed, in regard to many cases, is entirely against the assumption, and is as complete as negative evidence can well be: consequently, it must be admitted that even this hypothesis, in the present state of our knowledge, is founded on the error of assigning to the whole genus what belongs only to particular species; and it would be trifling with the reader to attempt to prove that this is still more certainly and strikingly true with regard to inflammation of the mucous membrane of the stomach and intestines; an affection which, in innumerable cases in which its existence is certain, clearly appears, on the slightest examination of the succession of events, to be an effect and not a cause." (*Dr. Smith*, p. 31.)

Dr. Tweedie is also opposed to the doctrines which teach that an altered and vitiated state of the blood constitutes the essence of fever.

"Not only is the condition of the blood changed in typhous fever, but, as a consequence of this morbid state of the blood, the secretions are more or less vitiated. Hence the clammy, disagreeable condition of the mouth, the depraved taste, the dry sordes on the teeth and lips, the brown or black incrustation of the tongue, the peculiar smell from the body, which is easily recognised by those who have much experience in fever; while the excretions are much more fetid than in any other disease of a febrile nature.

"These morbid changes are evidently produced by the action of the febrific poison on the brain and nervous system, and not by its primary operation on the fluids: in this view alone are the principles of the humoral pathology at all applicable to the phenomena of fever in general." (*Dr. Tweedie*, p. 50.)

From Dr. Burne, some of whose opinions we think are not likely to make many proselytes, he also differs.

"If the term adynamic fever, which, to borrow Dr. Burne's own definition, 'means a state of debility, from a depression or prostration of the powers of the nervous and muscular systems, not ordinary debility, as from loss of blood, or from wasting of the physical powers,' be intended to include the putrid, malignant fever of Sydenham; the slow, nervous fever of Huxham; the nervous fever of common language; the synochus, typhus mitior, and gravior of Cullen; the jail and hospital fever; the *fièvres essentielles* of the French; the epidemic of the Irish writers; the contagious of Bateman; the typhus of Dr. Armstrong; and the proper idiopathic, a common fever of Dr. Clutterbuck, the description of fever which he has given should have included all those various and opposite forms, which are in many respects very different in their nature, and require each an appropriate, and in some measure opposite, mode of treatment. But when a description of the symptoms of all these varieties is blended, with the view of showing that the adynamic form is the prevailing character of the fever of this metropolis, I feel it my duty to state that, however well Dr. Burne has described one variety, his description by no means applies to the ordinary fever of London." (*Dr. Tweedie*, p. 52.)

The frequent and formidable disease, in the investigation of which we are entering, cannot be understood, says Dr.

Smith, until clear and exact answers are obtained to the following inquiries.

"1. What is the series of phenomena which constitutes fever? 2. What are the particular phenomena which are common to all its varieties and combinations? 3. What is the order in which these phenomena occur in the series? 4. What are the organs, and what their states, upon which these phenomena depend? 5. What are the external signs of these internal states, or what are the indications by which their existence may be known? 6. What is the external noxious agent or agents, or the exciting cause or causes of the disease? 7. What is the particular remedy, or the particular combination of remedies, which is best adapted to each state of each organ?" (*Dr. Smith,* p. 33.)

Our first duty is to ascertain the concourse of symptoms, and the second to determine the order in which they occur.

"When these two points have been made out, what is essential and what adventitious, as well as what is the cause and what the effect, becomes at once clear and certain. But the difficulty lies in discerning, amidst the infinite diversity and contrariety of symptoms which the different modifications of fever present, when we may safely assure ourselves that we are in possession of all the essential phenomena. Our guide is invariableness of concurrence. If we can ascertain that a certain number of events invariably take place in every form and every degree of fever, these events will give us the particular phenomena which are common to all the varieties of the disease. If we can further ascertain that these events invariably concur in a certain order, we shall have discovered what events bear to each other the relation of cause and effect. And the establishment of this relation of events, this constant connexion with each other, this uniform antecedence and sequence, appears to me to be the only theory after which it is consistent with the principles of sound philosophy to search." (*Dr. Smith,* p. 36.)

If Dr. S. has endeavoured to establish this connexion, and has thus ventured, as we conceive with him, in a strictly philosophical sense to propose a theory, in doing so, he has carefully restricted himself to the attempt to deduce a legitimate conclusion from facts previously ascertained.

Chapter II. Dr. Smith proceeds to point out the various appearances presented by fever under different circumstances, and in different climates. If this fact, which it would scarcely be supposed could ever have escaped the notice of the most superficial observer, had been duly attended to by many writers upon the subject, the perplexities which have arisen from their attempts to draw a general description of fever from particular epidemics, or a few insulated cases, would have been entirely avoided.

"Something there is, however, which, amidst this astonishing diversity, preserves the identity of the disease so completely and so obviously, that there never has existed any dispute about that identity, under any aspect which it has hitherto been observed to assume; so that all physicians, without exception, unhesitatingly accord the name of fever to the mildest form of the common fever of this country, to the yellow fever of the West Indies, and to the plague of Constantinople and of Egypt." (*Dr. Smith*, p. 42.)

If three persons, each exhibiting an exquisite specimen of one of these several forms of the disease, were brought into the same ward of an hospital, the external aspect presented by each would be so different, that an unprofessional observer would probably be able to discover, in these modifications of the same malady, no common property; yet there is no physician who would not, in each case, instantly pronounce the disease to be fever.

"There must, therefore, be something that establishes the identity of the disease under this diversity of aspect. What is that something? Whatever it be, it must be common to all the varieties of fever. Thus we are led at once to the second inquiry which we proposed to keep before us in this investigation, namely, what are the particular phenomena which are common to all the varieties and combinations of the disease?" (*Dr. Smith*, p. 43.)

Fever, it must always be remembered, is not an entity, not a being possessing a peculiar nature; and the object of investigating it is not to discover in what such nature consists, or what it is that constitutes its essence; "but fever is a series of events, and the object of inquiry is to discover what the events are; what the events are that invariably concur in the series; and in what order they constantly succeed each other." (*Dr. Smith.*) It is not, however, the invariable concurrence of a particular number of events that is alone sufficient to constitute fever: to this must be added invariableness of concurrence, in a particular order.

"The order of events then is, first, derangement in the nervous and sensorial functions; this is the invariable antecedent: 2dly, derangement in the circulating function; this is the invariable sequent: and 3dly, derangement in the secreting and excreting functions; this is the last result in the succession of morbid changes." (*Dr. Smith*, p. 50.)

"Every one who has attentively studied the order of invasion of the symptoms, and more particularly those who have had personal experience of fever, must be satisfied that the brain and nervous system are early and primarily engaged in the febrile action; the disturbance in the brain is, in the beginning, simply functional, though it may, sooner or later, according to particular circumstances, assume an inflammatory character.

"The circulation next partakes in the disorder: there is generally, though not invariably, quick pulse and heat of skin, to which, as a consequence of the previous condition of the sensorium, succeeds a vitiated state of the secretions. Hence the furred tongue, thirst, depraved taste, and turbid urine, observed in fever." (*Dr. Tweedie*, p. 6.)

No other disease exhibits, says Dr. Smith, the same train of phenomena in the same order of succession. "In inflammation some of the phenomena are the same, but the order in which they concur is not the same; and this affords a clear and universally applicable mark of distinction between fever and inflammation." In inflammation, the earliest indications of disease that can be discovered have their seat in the affected organ itself. We admit the correctness of the distinction laid down by Dr. Smith between fever and inflammation; but it unfortunately happens that, in the great majority of cases, the practitioner cannot trace the different order in which the characteristic phenomena occur. He very rarely sees his patient early enough after the first attack of disease to enable him to determine, with any degree of confidence, whether fever or local inflammation constituted the primary malady. Dr. Tweedie observes, that it is important to remark that many patients admitted into the hospital had been, for a long period previous to the attack of fever, the subjects of incurable organic diseases, upon which fever had supervened.

"There is another description of cases which are not unfrequently received, viz. cases of neglected inflammation of some important organ, of which the fever is merely symptomatic. These cases are by no means easily discriminated from idiopathic fever, during the progress of which some organ has become inflamed: the obscurity arises from the impossibility, in most instances, of obtaining a correct history of the early symptoms; the difficulty, in all cases of complicated fever, being to trace out and determine whether the fever is primary, or symptomatic of some local affection." (*Dr. Tweedie*, p. 20.)

No speculative attempts are made by the authors to determine the proximate cause either of fever or inflammation. Dr. Smith very truly states, that what the physical and the physiological condition of the organs is, as contrasted with their condition in a state of health, has not yet been made out with regard either to fever or inflammation.

"What inflammation is beyond the series of events we are able to observe, we do not know; what fever is beyond the series of events we are able to observe, we do not know: we compare the events, and we see that they differ; and, since the use of names is to mark and to express differences, it is right to distinguish these different events by different terms. But though, in the present state of our knowledge, we are not justified in considering fever and inflammation to be the same, yet the close, perhaps the constant, connexion between them, is a fact of the utmost importance to be known, and requires to be incessantly before the view of the practitioner; and of this we shall have but too abundant evidence in the sequel." (*Dr. Smith*, p. 52.)

Out of the system of organs that are always affected in fever, some may be more and some may be less diseased; and it is easy to see how, from this diversity alone, the utmost variety may arise in the external characters of the disease. At one time the spinal cord and the brain may be intensely affected, and pains in the limbs, ferocious headach, early delirium, may occur. "Or, on the contrary, all the muscles of voluntary motion may be seized instantaneously with such a loss of energy, that they may truly be said to be paralysed." (*Dr. Smith.*) This statement is no doubt founded upon observation, but it has not fallen to our lot to witness any such "instantaneous" loss of muscular energy in fever cases. Dr. Tweedie expresses his surprise that, from the frequent occurrence of symptomatic inflammation of the brain in the more acute as well as chronic forms, that palsy so rarely follows. At another time the disease may seize with peculiar violence upon the organs of secretion, the digestive organs in particular.

"When the spinal cord and the brain are so violently affected that the patient appears to be struck with paralysis or apoplexy, the attention is not strongly drawn to the state of the mucous membrane of the digestive apparatus; to the nature of the secretions and excretions of which it is the source; to the temperature of the system, or to the condition of the circulation; because the affection of the nervous system being overwhelming, and all the other affections being comparatively trifling, it is natural that the former should, in a manner, absorb the mind of the observer; yet if the skin, the pulse, the tongue, the evacuations, are examined, all will be found to be in a morbid state, and that morbid state will bear a certain proportion to the affection of the nervous system." (*Dr. Smith*, p. 55.)

"There is another point connected with the state of the brain in fever, to which I wish to call more particularly the attention of those whose opportunities of treating the disease are limited, viz. that when some other organ becomes inflamed in the course of the febrile action, the symptoms are more or less obscured by the cerebral affection.

"It is in this way that symptoms in the chest, for example, may be entirely overlooked; and in the absence of cough, and of any disorder in the respiration, there is nothing to lead even to the suspicion that there is mischief going on. The condition of the brain is the cause of this obscurity, and it is in such cases of latent pulmonary disease that the stethoscope is of unquestionable diagnostic value." (*Dr. Tweedie*, p. 31.)

Retention of urine may be ranked among the paralytic affections depending on the condition of the brain in fever. In various diseases of the head, the bowels are with difficulty stimulated by ordinary aperients, and therefore the more active kinds are required.

"From an inactive state of the muscles concerned in the expulsion of the urine, accumulation in the bladder often takes place; so that, in all cases of severe sensorial disturbance, the region of the bladder should be examined at each visit, as I have often seen great additional irritation arise from this cause. I have known a practitioner thrown off his guard completely by the patient passing small quantities of urine unconsciously, which not unfrequently happens when the bladder is over distended. Appropriate measures should be adopted before such an accumulation takes place, as it not only proves a source of distress, but the sudden removal of so large a quantity by the catheter in the advanced stages of fever, is sometimes followed by an alarming collapse, from which it is not easy to rouse the patient." (*Dr. Tweedie*, p. 32.)

In fever, as in other diseases, the greatest diversity of symptoms may arise from different degrees of the same affection of one and the same organ.

"One degree of affection of the brain, for example, will occasion violent headach, constant watchfulness, great restlessness, a peculiar expression of the eye, and intolerance of light; in another there will be no headach, or none of which the patient will complain; there will be sleep, though it be disturbed and unrefreshing; there will be no peculiar expression of the eye, and no intolerance of light. By one degree of affection, the sensibility will be rendered preternaturally intense; by another, it will be totally obliterated: one will produce violent delirium, another only slight wandering, or unrefreshing slumber; one, violence requiring restraint; another, profound coma. In the circulating system the symptoms will alike vary. One degree will produce a quick, strong, and hard pulse; another, a quick, small, and feeble pulse; another, a slow and intermittent pulse. A similar diversity will be found in the temperature of the body: in one, the heat will be little changed; in another, it will be below the natural standard; in a third, it will be intense, and the organs of secretion and excretion will equally vary in the extent of their morbid changes." (*Dr. Smith*, p. 57.)

When to this variety are added diversities occasioned by various stages of the diseased processes that are going on in the system; by the previous state of the organs affected; by the reaction of the affected organs one upon another, producing innumerable and ever-varying combinations of different intensities of affection, in different sets of organs; and by the treatment to which the whole have been subjected, we cannot wonder if the symptoms of fever appear to be countless.

"That no two cases of fever can ever be precisely the same, and that it must be vain to seek for the common phenomena of the disease in the external symptoms, must now be obvious; and why success can never attend the search after these common phenomena in such symptoms as 'shivering, frequent pulse, heat,' must be equally manifest. These, as well as all other symptoms, depend upon the state of the organs." (*Dr. Smith*, p. 58.)

The division of febrile diseases into idiopathic and symptomatic, is condemned by Dr. Smith. It is liable, he contends, to the fundamental objection that the diseases included under the second section are not fevers, but inflammations. He admits no fevers but idiopathic fevers.

Upon the subject of simple or idiopathic fever, Dr. Tweedie speaks with some hesitation.

"I am aware that many distinguished pathologists not only doubt, but positively deny, the existence of what has been termed simple fever, that is, fever without evident symptoms of local inflammation. On this point, I may state that I have daily opportunities of observing cases, which correspond with the description of the simple fever of many writers, in which there is no preponderance of action in any organ that can be detected by symptoms; but when we recollect how often organic disease steals on, undetected by diagnostic signs, how much we are at times deceived by latent local diseases, the condition of the organs in what is termed simple fever, requires minute diagnostic investigation.

"Of the whole number of cases which occurred at the hospital within the period of this report, more than one hundred came under the description of simple fever; that is, the disturbance in the system was general: there was no evidence by symptoms of affection either in the head, chest, or belly.

"The character of this class of cases was, increased heat, accelerated pulse, thirst, and general functional disorder." (*Dr. Tweedie*, p. 26.)

Broussais, with his characteristic dogmatism, asserts "that a case of fever never has been, and never *will* be, seen, in which all the tissues of the body are equally affected."* In other words, that there is no such disease as simple or idiopathic fever. What other practitioners may have seen,

* Examen des Doctrines Médicales, &c. tome ii. p. 399. Paris, 1821.

or what we may in future see, we will not venture to determine; but we certainly have never yet seen a case of fever in which there was not sufficient evidence that the preponderance of disease was borne by some one organ of the body.

Dr. Smith deals very freely with the received nosological arrangements of fever.

"The more we investigate the subject, the more satisfied we shall become that continued fever is one disease, and only one, however varied, or even opposite, the aspect it may present; but that it differs in intensity in every different case, and that this, and this alone, is the cause of the different forms it assumes. Many of these diversities it would be frivolous to distinguish; some of them, on the other hand, it is of the highest importance to discriminate. For all useful and practical purposes, it is necessary only to arrange the different assemblages of symptoms into two great classes, the one comprehending the mild, and the other the severe, forms of the disease. All the forms that continued fever can assume, and all the individual cases that can occur under either, must be mild or severe, and therefore must readily find its place under one or other of these divisions. The only real difference in the disease being a difference in degree, it is proper that the principle of the division by which the varieties it presents are classified, should be founded on this, the only true distinction of which it admits." (*Dr. Smith*, p. 71.)

We should object decidedly to simplicity of arrangement, if it were obtained at the expense of accuracy; but we are convinced Dr. Smith is correct in this statement. Various other practical writers have arrived at a similar conclusion. As one example, we may cite Dr. O'Reardon, whose opinions upon this subject we lately extracted from his Report of the Fever Hospital of Dublin. "The divisions of fever (says Dr. O'R.) designated in our classic medical works by the appellations of Synocha, Synochus, Typhus mitior, Typhus gravior or putridus, Febris biliosa, Febris nervosa, and Febris maligna; and which Pinel terms Fièvre angiotenique ou inflammatoire, Fièvre meningo-gastrique ou bilieuse, Fièvre adenomeningée ou muqueuse, Fièvre adynamique ou putride, Fièvre ataxique ou maligne: all these divisions are, I venture to say, mere variations of one genus constituting fever; varieties produced by the diet, occupation, locality, temperament, habit of body, state of mind, or nervous condition of the patient; or by an alteration in his biliary or other secretions; or by the period of the fever, or its treatment; or by the season of the year, the climate, or constitution of the atmosphere."*

* London Medical and Physical Journal, August 1829, p. 172.

Dr. Smith adopts two words from the nosology of Cullen, and employs them merely to express differences of degree relative to one and the same disease. The mild degree he terms Synochus, by which is implied the ordinary form of fever in this metropolis, and even in this country. The severer form is designated Typhus. "Each will be found to present a distinct assemblage of symptoms; each will be found to depend upon a particular condition of certain organs; each will be found to require a peculiar treatment." (*Dr. Smith.*) To distinguish further important differences, which bear an important relation to practice, each of these two great classes is divided into two minor sections. Synochus into s. mitior and s. gravior; and typhus into t. mitior and t. gravior. Dr. Tweedie divides continued fever into simple, complicated, and typhus. Dr. Smith thinks that his principle of arrangement might be extended with advantage to the exanthemata, and all the forms of fever which have hitherto been known to exist, or which can arise.

In the third chapter, Dr. S. enters into a detailed account of the successive phenomena which constitute synochus mitior, and the indications afforded of disease in the nervous, circulating, and excreting systems. The progress of disease. consisting in progressive increase in the derangement of these functions, the phenomena of recovery, and in what circumstances depends the transition of synochus mitior into synochus gravior, are also described. The classification must be according to the different organs in which the several affections have their seat. Hence synochus gravior, with cerebral affection, subacute, acute; with thoracic affection, with abdominal affection, with mixed affection.

Having most accurately described the succession of phenomena in synochus mitior, Dr. Smith next enters upon the important subject of synochus gravior with cerebral affection, which occurs under two degrees of intensity: when the cerebral affection is moderate, it may be termed subacute; when great, acute. He particularly insists upon the utmost vigilance in watching the insidious approach of these cerebral affections. He who looks for intense pain, and suspects no cerebral affection, unless accompanied with this symptom, will be surprised at the sudden occurrence of new symptoms, which will at length open his eyes to the danger of the case. "The warning (says Dr. S.) was given, but the sign was not understood."

It is not uncommon for the most unequivocal and extensive changes of structure to take place in the brain and its membranes, without severe pain having ever been felt. Pain, however, though it be not great, is almost always present.

"Now and then no pain whatever is felt. Question the patient as much as you please, and he will tell you that he never has felt any pain. In this case giddiness is the substitute. Giddiness in the commencement and in the early stage of fever, is as certain a sign of cerebral affection as pain. Striking illustrations of this are afforded by several cases detailed in the pathology; by consulting which, the reader will see that precisely the same morbid changes take place in the structure of the brain, although nothing but giddiness be complained of, as occur in those which are attended with the acutest pain. The practitioner will therefore fall into a fatal error who is seduced into security because pain is absent; and who neglects the remedies proper for inflammation of the brain, because the patient complains only of giddiness. If giddiness be combined with pain, or alternate with it, which is not uncommon, the giddiness being slight if the pain be severe, and the pain being slight if the giddiness be distressing, it indicates a more severe affection than if either exist alone." (*Dr. Smith*, p. 98.)

The dull and heavy expression of the eye, when there is an accompanying cerebral affection, is greater than in the milder form of fever. There is usually a corresponding increase in the general sensibility. A loud noise is invariably distressing to the patient. Exposure to a glare of light and a loud noise may alone rapidly change a slight into the severest cerebral affection. As long as the pain, the giddiness, and the increased sensibility continues, there is invariably a want of sleep. At the close of this train of symptoms, delirium sometimes occurs. It is seldom violent or long continued; but, when present, is like the talking of a person during sleep, in a disturbed dream. The pulse, during all this time, may not be much quicker than in the mild form. These symptoms of prominent affection of the brain continue without intermission, and with little change, for several days. An entirely new train of symptoms then supervenes, which presents a striking contrast according as the patient is destined for life or death. These opposite symptoms, which are of so much consequence as guides to our prognosis, are minutely described by Dr. Smith. When synochus gravior is accompanied by acute cerebral affection, the history is precisely the same, excepting that the symptoms are more severe, and their progress quicker. The slow and intermitting pulse Dr. S. considers very characteristic of an exceedingly acute attack of cerebral disease.

"Whenever, in the onset of fever, a patient is found with intense headach, or intense pain in the back and loins, and a slow pulse, the physician ought to be greatly alarmed at the severity of the symptoms that are to follow; and if he do not take the most active measures to break the violence of the disease at this early period, it will be beyond all control in a day or two, and the patient will be dead before the fever is well formed in milder cases." (*Dr. Smith,* p. 109.)

The case of Dr. Dill is adduced as a proof that pain of the head is far from being the first symptom that occurs in the most intense cerebral attack. We recently attended a young lady who had apparently enjoyed the most perfect health until within a month of her death. She complained only of slight pain in the head, which usually occurred after dinner. Upon dissection, the dura mater covering the left hemisphere of the cerebrum was found extensively ulcerated, with thickened and hardened edges. In this instance severe cerebral disease must have been proceeding for some time, without any symptoms that are usually considered indicative of such an affection. Dr. ABERCROMBIE, in his valuable Treatise on the Pathology of the Brain, mentions many similar examples of the occasionally insidious progress of diseases of the brain. To illustrate each form of synochus which has been described, Dr. Smith relates several cases.

Dr. Tweedie makes the following important remarks upon affections of the brain in fever.

"Of the 521 cases, 114 had well-marked symptoms of severe cerebral affection, indicated by one or more of the following symptoms: pain, giddiness, sense of weight or fulness, watchfulness; and in the advanced stages, delirium, coma, spasms, or more rarely convulsions. This latter symptom was observed in four patients only, and of these, two had been previously subject to occasional attacks of epilepsy. One or more of these symptoms showed that the brain was seriously involved in the febrile action. The danger of the case depended on the extent of inflammation, the treatment which had been adopted, and its effects on the disease; for when the proper stage for active treatment had been allowed to pass over, the hopeless signs of neglected cerebral inflammation left little to be done but to pronounce the fatal issue of the disease.

"In a large proportion of the cases, the condition of the brain constituted only part of the danger, other organs being at the same time inflamed; for example, in 26 the head and chest, in 30 the head and belly, and in 14 the head, chest, and abdomen, were simultaneously affected.

"It appears that in 184 the brain was seriously involved in the febrile action; and, on referring to the table I have drawn up of the morbid appearances observed in the fatal cases, it will be seen that, in 37 out of 54, (the whole number examined,) the brain showed evident marks of the existence of previous inflammation.

"In fourteen of the fatal cases, there were no traces of any disease in the brain or its membranes. In these, destructive inflammation of some other organ, which had supervened in the course of the fever, was the immediate cause of death." (*Dr. Tweedie*, p. 28.)

In the next sections, Dr. Smith describes synochus gravior with thoracic and abdominal affections, and gives some cases as examples of these forms of disease. Under the head of Synochus gravior with mixed affection, Dr. S. includes those cases which are neither in an exquisite degree cerebral, thoracic, nor abdominal, but which at one and the same time afford the most exquisite specimens of all the three.

"From this account of the sense in which the term is employed, it must be obvious that it will include the severest cases that can occur. If a patient be affected with intense cerebral disease, he may be in great danger; but if he be affected with an equally intense thoracic disease, his danger must be doubled; and if to this be added an equally intense abdominal disease, it must be trebled. And accordingly these are just the cases which bid defiance to the most skilful and vigorous measures which the medical art can employ to control them; which seize upon their victim with a force which no human agency can resist nor counteract; which in malignant epidemics destroy life in a few hours or in a single hour, and in ordinary seasons in a few days." (*Dr. Smith*, p. 143.)

We doubt whether these mixed cases are usually the severest. It has appeared to us that in fever cases, where several organs were attacked, that neither was so intensely affected as when one organ alone bore the whole violence of the disease, and that consequently the danger was diminished. Dr. Crampton also expressly states that "where *a number* of important organs were pressed at the same time, as the brain, the lungs, and the stomach, the danger was less than when the whole force of the disease fell upon *one*, with undivided and concentrated violence. In such cases, provided patients were admitted at early periods, it was satisfactory to see the advantages resulting from an active and energetic practice, and how soon they became convalescent."*

*Of Typhus.* Under this term Dr. Tweedie includes,

* Medical Report of the Fever Department in Steevens' Hospital, from 1817 to 1819. By John Crampton, M.D.

"those fevers in which the brain and nervous system are early and severely affected, accompanied with symptoms denoting a morbid condition of the mucous membrane and skin, and a tendency to what is known by the term putrescency. This tendency is indicated by the condition of the blood, especially in the advanced stages; the crassamentum of which, instead of forming a firm coagulum, is loose, small in proportion to the quantity of serum, and so soft that it breaks readily on attempting to raise it, resembling in consistence half-boiled currant jelly. In some instances, I have observed that, when blood has been abstracted late in the disease, it scarcely coagulated at all." (*Dr. Tweedie*, p. 49.)

Dr. Burne has drawn a very good description of this fever, but he has given an impression that the simple typhus, or adynamic fever, is the general character of the common, continued, or epidemic fever of London. From this opinion Dr. Tweedie dissents, and in our opinion very justly. Dr. Smith considers that the difference between synochus and typhus arises entirely from a difference in intensity, but that still this difference produces a very important modification in the character of the disease: important because it materially affects both the safety of the patient and the nature of the remedies that are best adapted to rescue him from his danger. Typhus, like synochus, presents itself under two degrees of intensity, which, like those of the latter, Dr. S. designates by the terms mitior and gravior. All the important symptoms which belong to both are found in the same cavities, and relate to the same organs, as in synochus, and therefore must in like manner be divided into cerebral, thoracic, and abdominal. Dr. Tweedie states that typhous fever may be simple, that is, uncomplicated with inflammation in any of the organs, like the more common varieties of epidemic fever: however, it "very often," in its progress, becomes complicated with local congestion or inflammation, either in the brain, chest, or belly. For the term "very often" we should be inclined, from our own observation, to substitute "almost always:" we believe that cases of simple typhus, or of Dr. Burne's *simple adynamic fever*, both which expressions imply fever unaccompanied by organic inflammation, are extremely rare.

Dr. Smith has seen no example of typhus gravior in London. He has witnessed nothing bearing a tolerable resemblance to this disease, even as it is depicted by Cullen; much less as it is portrayed in the darkly vivid, yet apparently but too faithful colouring of Huxham. All the examples of fever which approach in likeness to the descriptions on record of typhus gravior which he has seen, have consisted of the mixed cases of typhus. They have been cases in which the brain, the lungs, and the intestines, were all simultaneously and intensely affected. Such cases Dr. Smith considers referrible to two classes; one in which the arterial action is excessive, the other in which it is oppressed, or rather overwhelmed.

"1. In the first, the patient lies insensible, with delirium perhaps so violent that he cannot be kept in bed without restraint; with extreme restlessness and constant watchfulness; with rapid and panting respiration; with a tender abdomen, perhaps with frequent and involuntary stools; a dry, black, and hard tongue; a quick, yet weak pulse; and the skin universally and pungently hot.

"2. In the second, he lies insensible, with a cold and dusky skin; with a swollen and livid countenance; with a heavy and oppressed respiration; with a pulse perhaps not to be felt, or, if distinguishable, either so rapid that it cannot be counted, so small that it is like a thread beneath the finger, and so weak that it is lost by the slightest pressure, or else slow, irregular, and intermittent. In this state, the patient is almost as completely paralysed as in apoplexy, and the attack is almost as rapidly fatal as apoplexy. It constitutes what has been called congestive fever. (*Dr. Smith*, p. 163.)

Fortunately these intense forms of disease are rare; they are witnessed only in solitary instances. Dr. S. combats with energy the dangerous notion that such cases form exquisite specimens of diseases of debility. Where, he asks, is the debility?

"'Not in the disease, for that is of giant strength; not in the patient, for remove, if you can but remove, a part of the load that oppresses him, and instantly an intensity of action will be set up in the whole system, perhaps as great as it is capable of exerting, and certainly greater than it is capable of sustaining without the most imminent danger. The brain is overwhelmed by the intensity of its affection; the energy that should animate the system, and of which it is the great source, is withheld: but that energy is suspended, not destroyed; and the debility which seems to be the result is not real, but apparent, not direct, but indirect. The giant that lies prostrate on the earth, mastered by superior power, has still a giant's strength, though he do not at that moment put it forth: give him but the chance of throwing off the load that keeps him down, and he will soon show you that he is not weak." (*Dr. Smith*, p. 164.)

We are convinced, with Dr. Smith, that no doctrine can be more fatal, if indiscriminately applied, and if taken as a guide for our general practice, than that which would lead us to attribute these congestive forms of fever to debility. But we are also convinced that cases do occur in which the smallest quantity of blood cannot be safely abstracted. Dr. Tweedie relates a case in point.

"In this patient, the description of fever was purely adynamic: the most remarkable features were, the greatest muscular prostration, with nocturnal delirium, so that she lay sunk in the bed, passing her stools involuntarily without the slightest pain, or any symptoms of local disturbance. It was necessary, in the very first stage of the disease, to administer wine and stimuli very freely; under which treatment she slowly, though eventually, recovered; but her convalescence was retarded by that peculiar swelling of the lower extremity which I have described. This lady certainly was saved by liberal doses of wine; and so great was the 'tendency to death,' that for forty-eight hours it was necessary to sit by her bedside with the finger on the pulse, and to administer stimuli whenever it appeared to become soft and compressible; in fact, the heart's action seemed to be completely under the control of diffusible stimuli.

" If such treatment were applied to cases of epidemic fever in general, I need not anticipate the result; or had antiphlogistic measures been adopted in the case of this patient, I can safely say that the abstration of a few ounces of blood, or even a brisk purgative, would have been instantly fatal. The necessity, therefore, for discrimination in the treatment of fever, is evident; for although much information and assistance may be obtained from the prevailing character of the disease, yet every individual case must be treated *per se;* with due deference to its particular and individual circumstances." (*Dr. Tweedie*, p. 53.)

We must pass over the observations Dr. Smith offers upon Scarlatina with but one comment. He states that "the depression of the nervous system so characteristic of synochus and typhus, is much less in degree in scarlatina. Neither the physical nor the mental debility is as great. In the whole attitude and manner of the patient, as well as in his own sensations, there is less prostration. The disease is more nearly allied to a pure inflammatory affection than either of the preceding forms of fever."

Such we believe to be a correct description of the ordinary character of scarlatina. But in this form of fever, as in every other, particular epidemics assume particular characters, and require a modification of the ordinary mode of treatment. The scarlatina which raged so extensively in this metropolis at the latter part of last year, was marked *from the commencement*, in many instances which fell under our own notice, by the utmost degree of mental and physical debility. In some of these cases in which even local bleeding was very moderately employed, the result was rapidly fatal; while in others, of an apparently similar nature, carbonate of ammonia and small quantities of wine were given in the early periods of the disease, with the most decided advantage. In two of the cases to which we refer, Dr. Macleod was consulted, and can bear his testimony to the benefit of this practice. Dr. Tweedie states that he knows of no treatment in the malignant form of scarlatina that is useful. "Bloodletting, or any kind of active measures, was out of the question." Dr. Smith also remarks, that the most exquisite specimens of congestive fever which he has witnessed have been those afforded by scarlatina, and that there is no disease incident to this climate which is more alarming, more beyond the reach of remedies, or more rapidly fatal.

*Of the Pathology of Fever.* Having very minutely and ably detailed the symptoms of fever, Dr. Smith proceeds to the pathology of the disease. The external appearances of the body after death, the morbid appearances in the head, thorax, and abdomen, are described by both our authors at much length; they each relate numerous occurrences in illustration of the various morbid changes which occur in the different organs. Dr. Smith gives an example of the modifications which take place when fever proves fatal in the state of gestation. "If fever," he remarks, "attacks during pregnancy, there is the greatest possible danger of miscarriage, and the great majority of those who miscarry die." This observation is consonant with our own experience: we remember the late Dr. Thynne, to whose practical ability we are happy to offer our meed of respect, used to state in his lectures, that he never knew a woman miscarry from the shock of *any* acute disease who did not die. Having given many cases for the purpose of exhibiting a complete view of the pathology of fever, Dr. Smith briefly states the general conclusion to be derived. The account of the pathology of fever is the history of inflammation, and the description of the individual changes that take place in the organs that constitute the febrile circle, is an enumeration of various products of inflammation which are formed within them. There is scarcely a fatal case of fever which does not afford, in one or other of the organs of that circle, more inflammatory product; there is no considerable number of fatal cases which does not furnish a specimen of every inflammatory product. The same inference must be drawn for the statements made by Dr. Tweedie, and indeed it is now almost universally acknowledged by all pathologists, that proofs of inflammation are found in one or more organs, in most cases of fever which terminate fatally. Neither Dr. Smith nor Dr. Tweedie, however, assert that inflammation alone constitutes the state of fever. They are particularly careful in pointing out the differences that exist between these two affections. Dr. Smith is not willing to admit "that the danger of the patient is always in exact proportion to the degree of the inflammation," while Dr. Tweedie is of opinion that "the danger of the patient is always in proportion to the severity of the inflammation, and to the importance of the organ implicated." We must agree with Dr. Smith that now and then the intensity of the nervous affection in fever is so great, and so rapidly destructive of life, that there is no time for an inflammatory process to be set up, much less for an inflammatory product to be formed.

"The patient is struck dead as if by lightning, or by Prussic acid, or by apoplexy. In this country, he does not actually die as instantaneously as he might be destroyed by the electric fluid or by poison, although there are countries, seasons, and particular spots, in which the concentration of the febrile poison appears to be sufficiently great to extinguish life instantaneously; and even in this country, life is sometimes destroyed by a stroke of fever as rapidly as it is by a stroke of apoplexy, when the latter does not prove fatal in the first few hours." (*Dr. Smith*, p. 326.)

In these cases the internal organs after death exhibit no signs of inflammation, unless vascularity be inflammation. The organs which in ordinary cases are inflamed, are in these cases filled with blood. Upon the subject of the pathology of the fluids in fever, it is confessed that little is known. Some experiments, however, Dr. Smith informs us have been undertaken by Mr. Cooper upon an extensive scale, from which it is hoped our scanty stock of information upon this point of pathology may be increased. That deviations from the state of health, and some of them of great importance, do take place in the fluids, and especially in the blood and the urine, is ascertained. What they are, with what degree of constancy they occur, how far they are respectively connected with the cerebral, the thoracic, the abdominal, and the mixed affections, with different degrees of intensity in these affections, and with different stages of their progress, Dr. Smith hopes at no distant period to lay before the public.

The relation between the phenomena of fever, or the theory of the disease, is discussed by Dr. Smith in his seventh chapter. The differences between fever and inflammation are particularly pointed out, and it is shewn that

"The state of the system, in the primary attack of fever, and the state of the system in inflammation, do not appear to be identical. The truth is, that we do not know what the real state of the system is in either case, but we see that the phenomena of the one differ from those of the other; to conclude, therefore, that the states are the same is not a sound induction. While, then, we are constrained to admit that we know nothing of the nature of the primary affection of the nervous system in fever, the closest consideration of all the phenomena alike constrains us to conclude, that that affection is peculiar and specific.

"This peculiar and specific affection appears to be much more analogous to the condition into which the nervous system is brought by the application of certain poisons, than to that which is proper to pure inflammation. The more closely and extensively the subject is investigated, the more clear and satisfactory the evidence becomes, that the great primary cause of fever is a poison, the operation of which, like that of some other poisons, the nature of which is better understood, and the action of which has been more completely examined, is ascertained to be upon the nervous system. How these poisons act upon the nervous system we do not know, nor can we possibly know, as long as we remain so profoundly ignorant of the nature of the action of the nervous system in the state of health.

"It may be considered then as established, that the primary morbid condition of the body, in fever, consists of an affection of the nervous system, which there is reason to believe is of a peculiar and specific nature, although that nature be at present wholly unknown." (*Dr. Smith*, p. 336.)

The second event that takes place in the morbid series constituting fever is inflammation, but it must always be impressed upon the mind of the practitioner that the inflammatory state in fever is modified by the peculiar affection of the nervous system.

"Inflammation does not lose its nature by being combined with that peculiar affection of the nervous system which converts it into fever; it only modifies its state: the remedies proper for fever do not differ from those which are effectual in inflammation; they only require to be modified in accordance with the modified nature of the disease. He who believes fever to consist of an affection of the nervous system alone, every other affection that may be combined with it being accidental, will rarely think of using the lancet: he who believes fever to consist of inflammation alone, and overlooks the presence of the nervous affection, will be apt to carry the employment of the lancet too far: he alone who embraces the view of both, brings within his own all the phenomena: he alone adopts a sound theory of the disease, and we now see that he alone is likely to be led to a sound practice." (*Dr. Smith*, p. 342.)

We can best refer to the fifth chapter of Dr. Tweedie, and the eighth of Dr. Smith, for many interesting remarks upon the causes of fever.

*Of the treatment of Fever.* We shall first give the subtance of Dr. Smith's opinion upon this most important part of the subject. He believes that the only morbid condition of fever, of which we have any knowledge, and over which the medical art has any control, is that of inflammation. Again, however, he deems it necessary to repeat that febrile inflammation and ordinary inflammation are not identical, and that the difference between the two affections is such as to require a very considerable modification in the treatment appropriate to each. Although inflammation be not the primary febrile affection, as far as regards the order of events, yet it is, at least, the primary affection, as far as regards the treatment, if it be not the sole affection that admits of treatment. The remedies proper for febrile inflammation do not differ from those which are adapted to ordinary inflammation, but they differ materially in the mode in which they ought to be applied, and the extent to which they ought to be carried.

"They can be understood neither in their mode nor measure, until the following questions are determined; namely, what is the precise object that should be aimed at in the treatment of fever? What is it which it is most important to do, and which it is in the power of the medical art to accomplish? (*Dr. Smith,* p. 376.)

Fever, says Dr. Smith, cannot be cured instantaneously; but, if it come early under the care of the physician, and he know with promptitude and decision at what to aim, he will rarely fail in his efforts to secure this object. Since the various forms or type of fever differ in nothing but the degree of their intensity, in detailing the treatment it will be necessary only to state, first of all, the remedies which are appropriate to the disease; and, secondly, the modification of these remedies which may be required by the different degrees of intensity in which it is commonly found to exist. The common continued fever of this country, in its mildest form, requires little or no treatment. There is no affection of any organ intense enough to need the application of a powerful remedy. Confinement to the bed, abstraction of stimuli, fever diet, a Calomel purgative at night, followed in the morning by Castor oil, constitute the whole treatment required. Whenever the fever passes beyond this its mildest form, it becomes a serious disease, and is never for a moment to be trifled with. When the mildest case of fever passes to a severer form, the event that happens, as has been shewn by the preceding pathology, is inflammation, rising in degree and increasing in extent, or both, in porportion to the intensity of the febrile affection. To this general law there are few exceptions. The object to be aimed at in practice, then, is to prevent or remove inflammation. If this object be not accomplished, death will take place at last, in consequence of the destruction of the organs by the process of inflammation. The grand, nay almost the only, remedy recommended by Dr. Smith is bleeding. Bleeding, he says, cannot be performed too early in fever. "The very first moment of excitement, could that be discovered, is precisely the moment when the employment of this powerful remedy would produce the greatest effect." When inflammation has actually come on, there is then not a moment to be lost. Until the inflammation is subdued, blood must not be taken; and when this is done, nothing remains to be done. The vein must be allowed to flow, and it must be opened again and again, until this object is secured. To attempt to measure the quantity of blood abstracted by drachms or ounces is wholly vain, because, if the remedy be properly employed, the quantity will vary in every individual case. After all, the quantity of blood it is necessary to abstract is not large, for smaller bleedings will subdue febrile sooner than pure inflammation. If, after the abstraction of sixteen ounces of blood at the commencement of the attack, the vascular excitement be not completely subdued, in the course of three or four hours the same quantity must be taken again; and if the next morning the excitement continue, it will probably have already passed into inflammation, and, therefore, the vein must be once more opened, and the blood allowed to flow until the pain, wherever seated, be entirely removed. Local bleeding may often complete what the lancet commenced. A due impression having been made upon the inflammation by bleeding, purgatives of Calomel and Rhubarb, followed by Castor oil and the Senna draught, are to be given, to produce three or at most four stools in the twenty-four hours: beyond this, no advantage is obtained by purging.

"Cold sponging, if the skin be hot; acidulated drink, if there be thirst; perfect quiet, a dark room, a silent nurse, affording prompt attendance, with a noiseless step, a cheerful countenance, and no words; this, together with three teacupsful of thin arrow-root or gruel, in the twenty-four hours, given in divided portions, at intervals of about two or three hours, comprises all else that will be required, or that will be useful, until the period of convalescence.

"Such is the simple, but most efficient treatment appropriate to the common fever of London, and its neighbourhood, (and I do not speak of the treatment proper for any forms of the disease as it exists elsewhere, and which I have not seen,) in its ordinary degree of severity." (*Dr. Smith*, p. 387.)

In a note Dr. Smith observes, that it would be trifling to spend any time in discussing the merits of saline, refrigerant, diaphoretic, antimonial, medicines, &c. If the proper treatment have not been applied, and the disease from neglect or mismanagement have been allowed to take its course, symptoms of typhus or adynamic fever arise, for the product of every fever at a certain stage of its process is adynamia. But now bleeding relieves no symptom; it increases some; and the inference is that bleeding is a most inefficient and dangerous remedy in fever.

"Of what avail can bleeding be, when the patient is brought into the condition which first excites alarm, in the case here supposed? The blood is no longer in its vessels; it is beneath the membranes, or in the ventricles, or at the base of the brain; the inflamed capillaries have done their work upon the cerebral substance and upon its membranes; and have left proof enough of their activity, in the thickening of the one, and the softening or the induration of the other. What can bloodletting do in this stage of the organs? What can shaving the head and applying cold do? What can blisters do? What can purgatives do? And above all, what can wine do? Nothing can be done; at least, nothing effectually or certainly." (*Dr. Smith*, p. 390.)

If bleeding in this state be deemed prudent, the greatest caution must guide the use of the lancet. To decide in such a case requires the nicest discernment. Instead of bleeding, under these circumstances, the proper remedy may be the very reverse. A stimulus may be requisite. The powers of life may be so exhausted by the inflammatory excitement, that, unless aid be brought to them, they will be overpowered, and sink. This is precisely the condition, and, perhaps, it is the only condition under which stimuli are really beneficial in fever. Of all stimuli, wine or brandy is the best. If the stimulus be capable of doing good, some improvement in the symptoms is commonly perceptible in a few hours after it is first administered. No certain indication for the administration of wine can be drawn from one or two symptoms alone. There is an aspect about the patient, an expression in his countenance and attitude, in the manner in which he lies and moves, that tell the experienced eye when it is probable that a stimulus will be useful. There is a condition of the system in which an opiate puts a stop to a state of exhausting agitation and restlessness, procures sleep, and lessens delirium. But no kind of opiate ever proves beneficial when the chin is very hot, the tongue very dry, or the general motions and actions of the patient are violent. When the inflammatory action has terminated in some change of structure, probably accompanied with as copious effusion as indicated by the symptoms detailed under the cases of cerebral affection, advantage is sometimes obtained by affecting the system with mercury. Two grains of calomel with half a grain of opium should be given every three, four, or six hours. Some modification of the more powerful remedies will be necessary, as the prominent affection may have its seat in the brain, the lungs, or the intestines. When the attack commences with severe cerebral affection, the bleeding must be proportionally large, and early as it is copious. The "cold dash," when added to the use of the lancet, forms, by the combination, a treatment so powerful and efficacious, that it might render death from the acutest cerebral inflammation as rare as recovery is at present.

The mode of applying it is to pour upon the naked head of the patient a steady but continued stream of cold or iced water, from a wateringpot without the rose, the stream being made to fall as nearly as possible upon one and the same spot. At first the elevation must be slight, for the shock is too violent if the stream be poured at once from the highest point. "Employed as a remedy, there is no degree of burning heat which the animal economy is capable of producing, no intensity of vascular action, and no violence of pain, that can resist its continued application." Three or four repetitions will commonly suffice to subdue the most intense cerebral affection. In the case of Dr. Dill, the relief it brought was instantaneous and most complete.

In the severe bronchial affection of fever, bloodletting is of little avail. It weakens the patient, without making a decided impression upon the disease. Tartar emetic seldom fails, if exhibited with promptitude and decision. It should be given in doses of two grains dissolved in an ounce of water, and repeated every second, third, fourth, or sixth hour, according to the severity of the case.

General bleeding has but little influence over the inflammation of the mucous membrane of the intestines, which forms so constant and formidable a part of the organic affection in fever. If early employed, it may prevent the affection from occurring; but, when it has supervened, large bleedings are out of the question, and even small and repeated bleedings are not as effectual as leeches. When the purging is considerable, Hydrargyrum cum Cretâ with opiates, or opiate enemas, are useful. If there be constipation, small doses of Castor oil should be given. A few remarks are made upon the treatment of Scarlet Fever; and Dr. Smith concludes his volume by describing the appropriate treatment during convalescence.

The leading principles of treatment maintained by Dr. Tweedie are so similar to those supported by Dr. Smith, that it will be unnecessary for us to follow him closely through his remedial plan. We shall confine ourselves, therefore, to a few passages from Dr. T., some of which are extremely valuable, inasmuch as they caution the practitioner against the excessive employment or misapplication of the most powerful and necessary part of the required treatment. From his own observation, Dr. Tweedie can bear testimony to the practical import of the following doctrine, as applied to fever.

"The aged, infirm, and habitual free livers, in all diseases, bear bleeding ill. But, besides these more familiar classes, there is another, in which phlebotomy must be cautiously and sparingly practised.

"It consists of men, perhaps not above the middle age, whose minds and bodies, either from the circumstances in which they are placed, or from a natural ardor of temperament, are unceasingly taxed to the very utmost of their powers. With this class of persons, and medical men themselves too frequently belong to it, we must deal tenderly, or the mischief will speedily be irretrievable.

"It is also a well-established fact, that in some epidemics, and even at particular seasons, fever is not only more fatal, but does not bear bloodletting so well as at other times. We also know that in complicated fever the local symptoms vary in degree, and therefore require the discriminating hand of experience to apply, with advantage, a modification of this class of remedies.

"The experience of epidemic puerperal fever has shown that, though this severe, and often fatal, disease generally depends on inflammation of the peritoneum, and is most successfully treated by the early and free abstraction of blood, and other antiphlogistic measures, yet in some epidemics, or even in sporadic cases, these measures would be speedily destructive. This is owing not so much to any variation in the symptoms in the disease, as to some unexplained state of the system, at certain periods when puerperal fever is prevalent." (*Dr. Tweedie*, p. 173.)

If local bleeding is required, Dr. T. considers cupping very superior to leeching. The blood is drawn more rapidly, and the patient feels less fatigued, while the quantity can be regulated according to its effects. Both authors agree that inflammation of the mucous membrane of the bowels, and bronchitis, appear to be little controlled by general bleeding.

"When it was necessary to apply leeches when the powers of the patient were much sunk, I have seen almost fatal sinking from the profuse hemorrhage from the orifices. This hemorrhagic tendency is not peculiar to fever, as it occurs occasionally in other affections; but when it takes place from the abdomen, where pressure cannot be effectually applied, I have known it followed by fatal consequences.

"The practice of applying leeches late in the evening, and covering the belly afterwards with a hot poultice, without carefully watching the patient, should, if possible, be avoided; and when profuse bleeding cannot be restrained by lunar caustic, a fine needle should be passed under the bleeding orifices, and a ligature twisted round in the usual way." (*Dr. Tweedie*, p. 180.)

*Emetics.* "When the excitement is moderate, when there is no organic inflammation, and when the patient is young and vigorous, I think the shock given to the system by the effort of vomiting, is decidedly useful, by determining powerfully to the surface, and stimulating the organs of secretion.

"If, however, there be considerable vascular action, and more especially any local determination, the operation of an emetic is decidedly injurious. A moderate bleeding, under such circumstances, is the more judicious practice. In feeble subjects, or when the system is much lowered, they should never be employed." (*Dr. Tweedie*, p. 181.)

*Purgatives*, in some mild cases of fever, without any local determination, was sufficient to subdue the disease.

"From the opportunity I enjoyed of witnessing the efficacy of purgatives in the treatment of fever, while I held the appointment of physician's assistant to Dr. Hamilton, in the Royal Infirmary of Edinburgh, I am convinced that, next to judicious bloodletting, there is no class of remedies so decidedly useful." (*Dr. Tweedie*, p. 184.)

Indiscriminate purging, Dr. T. conceives to be equally injurious as indiscriminate bleeding. He is certain that much harm has been done by continuing the daily administration of purgatives in tedious cases of fever.

"Another class of cases in which active purging is improper, is subacute bronchitis, which is generally relieved by expectoration. Nothing is more likely to diminish this salutary process than the injudicious administration of purgatives. In such cases, therefore, the more mild aperients, so as to evacuate the bowels thoroughly without acting on the exhalents, were preferred." (*Dr. Tweedie*, p. 184.)

Mercury is administered in fever with three indications, as a purgative, an alterative, and to subdue inflammation.

"When mercury is employed externally, the best plan is to apply two scruples of mercurial ointment to the axilla, where absorption goes on very rapidly: this mode is more cleanly, and less fatiguing to the patient, than friction.

"The combination of calomel and opium, in inflammation of the chest and abdomen, is a remedy of great power, and I can speak with confidence in its favor." (*Dr. Tweedie*, p. 186.)

This remedy, however, will never supersede bloodletting, when the powers of the patient are able to bear it.

"It is important to avoid, if possible, the full mercurial action, because, when this occurred, I observed that great weakness ensued, which tended much to render the convalescence exceedingly tedious, and often imperfect. I have also occasionally witnessed very troublesome sloughing of the mouth, and even perforation of the cheek, which ultimately proved fatal, from the administration of mercury to children in fever." (*Dr. Tweedie*, p. 187.)

Saline medicines and diaphoretics are considered useless. Dr. T. has great confidence in tartar emetic, in idiopathic as well as symptomatic pulmonary inflammation.

As *Refrigerants*, the mineral acids, especially the oxymuriatic, are preferred. In the early stages they check the thirst and heat of skin, and in the more advanced, particularly when the fever approaches the typhoid character, their antiseptic powers render them decidedly useful.

*Narcotics* must be cautiously employed.

"When there has been much excitement in the brain, an opiate will certainly do harm. Even though the cerebral symptoms have passed off, and the patient complain only of restlessness and want of sleep, its advantages are doubtful. If it be given in such cases, a full dose of solid opium, combined with calomel, is the safest plan of administering it, while the scalp is enveloped in a cold lotion. Sometimes small opiates, repeated at short intervals, answer better than a full dose; for instance, eight or ten drops of the Liquor Opii sedativus in Camphor mixture, or in a saline aperient draught, every three, four, or six hours, carefully watching its effects." (*Dr. Tweedie*, p. 189.)

The acetate of morphia is recommended. It is less stimulating, and consequently less injurious should it fail to induce sleep.

*Cold.* The advantage of the free admission of cool air in the treatment of fever, is incalculable. Dr. Tweedie never saw the cold affusion, as recommended by the late

Dr. Currie, of any use.. In some instances it was injurious. He speaks highly of the effects of the cold dash.

*Treatment of Typhus Fever.* As a general rule, Dr. T. states that this form of fever neither requires nor bears phlebotomy.

"The rapidity with which the blood flows from the vein, and its appearance when drawn, form a very good criterion of the propriety of its abstraction in typhus fever. If, instead of pouring in a continued full stream, it come in drops, notwithstanding the vein has been well opened; when the blood coagulates slowly, the crassamentum being at the same time soft and easily broken, we may rest assured that the system will not bear bloodletting." (*Dr. Tweedie,* p. 194.)

Purgatives also must be employed with much caution in this form of fever. Daily inspection of the stools in typhus fever is indispensable. When there was any appearance of hemorrhage, the aperient medicines were immediately suspended.

Some judicious remarks are made by Dr. T. respecting the use of wine, and the management of the period of convalescence.

In the ninth and last chapter of his work, he treats briefly of Scarlatina, its varieties, of the epidemic scarlatina of 1828-9, and of its pathology and treatment.

If previous writers upon the subject of fever had pursued the path of investigation which has been so judiciously adhered to by Dr. Smith and Dr. Tweedie, the practical information we should have obtained must have been much more extensive. How many volumes have been written, and how much talent wasted, in the fruitless search after the proximate cause or essence of fever? In medicine, as well as in natural philosophy, it is sufficient for all practical purposes that we should be acquainted with the sensible phenomena, although the causes which produce them may escape our closest scrutiny. *Sufficit si quid fiat intelligamus, etiamsi quomodo quidque fiat ignoremus.* Hippocrates in medicine, and Newton in philosophy, confined themselves to the close observation of facts which were evident to the senses, without pretending to search into the proximate or hidden principles upon which the laws of disease or nature are founded. Dr. Smith and Dr. Tweedie have kept as closely as possible to a practical investigation of the highly important subject upon which they have had so much experience; they have encumbered their works with no visionary speculations, no flights of fancy, and hence their great value to the practical inquirer. Without instituting any invidious comparisons between the two works, we may observe that Dr. Smith, from the size of his book, has been enabled to enter more fully than Dr. Tweedie has done into the history of the chain of morbid phenomena which constitute febrile diseases, of which he has given a most masterly description. From the arrangement Dr. S. has adopted, frequent repetition of the same facts and doctrines was almost unavoidable. He appears, indeed, to be unconscious of the perspicuity and force of his diction, and often reiterates opinions and arguments which he had before fully impressed upon the attention of his reader. We admire the fluency and animation of his style, but regret it is not more concise. With respect to the treatment recommended, we are of opinion that Dr. S. urges the employment of the lancet with too few precautions and limitations.

Dr. Tweedie's book comes before us with less pretensions than that of Dr. Smith, but it affords ample proofs of accurate observation and practical judgment. It would be unjust to the authors, and to our readers, if we did not very strongly recommend the attentive perusal and study of both these volumes. The numerous cases detailed in them are highly interesting. In conclusion, we must be allowed to express our regret and astonishment that the name of Armstrong is not once mentioned, although some of his doctrines have not been forgotten.

### COLLECTANEA.

Floriferis ut apes in saltibus omnia libant,
Omnia nos, itidem, depascimur aurea dicta.

